# Specific characterization of regional storage fat in upper and lower limbs of young healthy adults

**DOI:** 10.1186/s40064-015-1181-6

**Published:** 2015-08-08

**Authors:** Junichiro Yamauchi, Toshiyuki Kurihara, Maki Yoshikawa, Sadayoshi Taguchi, Takeshi Hashimoto

**Affiliations:** Graduate School of Human Health Sciences, Tokyo Metropolitan University, 1-1 Minami-osawa, Hachioji, Tokyo, 192-0397 Japan; Future Institute for Sport Sciences, Tokyo, Japan; Research Center in Back, Neck, Other Joint Pain and Human Performance (BNOJPH), Faculty of Associated Medical Sciences, Khon Kaen University, Khon Kaen, Thailand; Department of Sport and Health Science, Ritsumeikan University, 1-1-1 Noji Higashi, Kusatsu, Shiga 525-8577 Japan

**Keywords:** Magnetic resonance imaging (MRI), Bioelectrical impedance analysis (BIA), Upper arm, Thigh, Body composition

## Abstract

This study aimed to determine the specific characterization of regional storage fat in the upper limb as compared to the lower limb of young healthy adults. The regional storage fat and skeletal muscle in upper and lower limbs were obtained by magnetic resonance imaging (MRI) and multifrequency bioelectrical impedance analysis (BIA). For MRI measurements, images at the continuous anatomical cross-sectional areas of subcutaneous adipose tissue and skeletal muscle in the upper arm and thigh were selected for the analysis. Values measured by MRI were larger than those measured by BIA. MRI data showed that the percentage of fat was significantly higher in the upper arm compared to the thigh in both men and women. This study suggests that BIA results in a significantly different estimation of the whole body and limb composition when compared to MRI and that MRI is useful to determine regional specificities in the limb composition. From these quantified evaluation, we found significantly large amount of regional storage fat in upper limbs of young healthy adults, especially women.

## Background

Modern lifestyles increase the prevalence of metabolic syndrome, which is associated with increased body fat. An increase in adipose tissue and decreased muscle mass causes decreased peripheral uptake of glucose and insulin resistance (Jocken and Blaak 2008; Roubenoff 2000). Metabolic syndrome contributes to atherosclerosis, causing reduced blood flow in the limbs. To maintain a good health status and understand the chronic metabolic responses in our body, measuring body composition is important (Lohman 1992). There are several ways to assess our body composition. Classical methods used to estimate human body composition include underwater weighing, body-water dilution, and body-potassium measurements, which have all been used in studies of human energy requirements and obesity (Duren et al. 2008; Sohlstrom et al. 1993). The bioelectrical impedance analysis (BIA) method is widely used to estimate human body composition in both clinical and practical fields because it requires minimal operator training and the results are available immediately. BIA offers a valid method for assessing body composition (Gray et al. 1989; Segal et al. 1988) and is an alternative to using dual-energy X-ray absorptiometry (DEXA), the “gold standard” reference method, for assessments of regional body composition (Anderson et al. 2012; Esco et al. 2014; Leahy et al. 2012; Shafer et al. 2009). Multifrequency BIA would be needed for appendicular lean mass estimation because predictions of arm and leg lean mass are more accurate at low and high frequencies, respectively (Anderson et al. 2012); however, it is still unclear whether whole body composition measured by BIA is equivalently estimated to the limb composition.

The scientific research community has commonly utilized magnetic resonance imaging (MRI) to evaluate lean muscle and adipose tissues due to the accuracy of measuring by contiguous MR slices. MRI offers an accurate and non-invasive means to assess the size and volume of regional muscle and fat (Commean et al. 2011). When MRI was used to quantify regional adipose tissue or skeletal muscle in humans, most studies measure a single cross-sectional area (CSA) image of abdominal or thigh (Cotofana et al. 2010; Lee et al. 2004). In this regard, there are limited data reporting analyses of the percentage of fat in upper limbs by MRI. A study with ultrasonic techniques has shown the age-related changes in CSA of fat and muscle of limbs during the growth period (Kanehisa et al. 1994). Therefore, the morphological assessment of the upper limb as well as the lower limb is important to determine the regional specificities of the body composition, and can provide useful information to advance our understanding of local limb morphology, which may provide new information to prevent metabolic and musculoskeletal disorders. Over the past years, the functional differences of the upper and lower limbs that occurred in the process of human evolution have been discussed; however, only few researches have investigated the morphological differences of the upper and lower limbs. We hypothesized that a specific characterization of regional storage fat and skeletal muscle in the upper limb could be identified as compared to the lower limb of young healthy adults. To solve this question, both BIA and MRI were used to quantify regional adipose tissue and skeletal muscle in vivo. Therefore, in this study, using the CSA image selected from multiple imaging protocols by MRI and separated limbs data from BIA, the human limb composition was determined and compared between the upper and lower limbs. The aim of this study was (1) to investigate whether the fat percent of limbs was equivalently estimated by using MRI and BIA, and (2) to determine the regional storage fat in upper and lower limbs of young healthy adults.

## Methods

### Subjects

Twenty-two young sedentary subjects volunteered, and their physical characteristics and anthropological parameters were shown in Table 1. This study was approved by the research ethics committee of Ritsumeikan University in accordance with the Declaration of Helsinki. All subjects were informed of the possible risks of the experimental procedures and gave their written informed consent before the investigation.

**Table 1:** Physical characteristics and anthropological parameters of men and women

	Men (n = 11)	Women (n = 11)
Age (years)	22.0 ± 3.0	23.0 ± 4.0
Height (cm)	173.8 ± 6.1	159.3 ± 4.6*
Body mass (kg)	64.5 ± 5.0	51.8 ± 5.3*
Body mass index (BMI)	21.4 ± 1.7	20.7 ± 1.6
Whole body fat (%)^a^	15.8 ± 0.03	27.9 ± 0.04*

Values are presented as the mean ± SD. * p < 0.05 vs. men.

^a^Percentage of whole body fat was measured by BIA.

### Experimental procedure

#### Bioelectrical impedance analysis (BIA)

For BIA measurements, the eight-contact electrode system with multiple impedance frequencies (InBody720, BIOSPACE Co., CA, USA) was used. The device can measure segmental impedance at multiple frequencies of five body parts (arms, trunk, and legs) at six frequencies (1, 5, 50, 250, 500, and 1,000 kHz). While a conventional BIA device needs to adjust the body composition from individual information such as the age and gender, this device can calculate the body component only from the value of the current impedance regardless of race, age and gender (Cha et al. 1995; Gibson et al. 2008; Kim and Kim 2013). The device uses eight tactile electrodes: two in contact with the palm and thumb of each hand, and two contacting the anterior and posterior aspects of the sole of each foot. During measurement, subjects stand upright with outstretched arms without touching their sides. They also wore light clothing and removed all metal items that could interrupt the electronic current during the measurements. Before the measurements, the subjects were asked to urinate just before the measurement, then rest on a chair for at least 5 min. Reproducibility of BIA measurement was evaluated using intra-class correlation coefficient (ICC) in four subjects. ICC(1,3) values of three successive trials for fat percentage of whole body, upper and lower limbs were 0.978, 0.987 and 0.977, respectively. Inter-day reliability was evaluated in ICC(2,1) values for fat percentage of whole body, upper and lower limbs were 0.969, 0.950 and 0.967, respectively.

#### Magnetic resonance imaging (MRI)

**Fig. 1:**
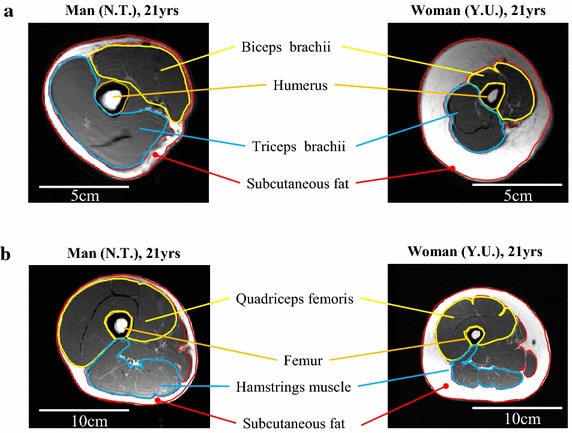
Representative axial magnetic resonance images of the *upper arm* (**a**) and *thigh* (**b**). *Left column* showing the man and *right column* showing the woman. The location of the image of the *upper limb* is at the level of the largest anatomical cross sectional area (ACSA) of the *upper arm* muscle. That of the *lower limb* is the ACSA at the level of 50% of the *thigh*. Subcutaneous fat, triceps brachii muscle, biceps brachii muscle, quadriceps femoris muscle, hamstrings muscle, humerus, and femur are outlined.

For MRI measurements, subjects were laid supine on the examination table of a 1.5-T MRI system (SignaHDxt 1.5T, GE Healthcare UK Ltd, Buckinghamshire, England) used in our previous study (Kurihara et al. 2014). The axial T1-weighted spin echo MR images (TR = 300 ms, TE = 10 ms, FOV = 240 mm, Matrix = 256 × 256, 2 NEX, 10-mm slice thickness, no gap) of the upper arm and thigh were acquired using an 8-ch body array coil. The MRI scanned from the head of the humerus to the styloid process of ulna and radius for the right arm, and from the greater trochanter to the knee cleft for the right thigh. Scannings for upper arm and thigh were executed separately and the subjects were repositioned in order to set the scanning limbs in the center of the magnet. During measurement of the lower limb, the image at the midpoint of the thigh was selected because the anatomical cross-sectional area (ACSA) of the muscle is the largest at this point (Cotofana et al. 2010). For measurement of the upper limb, the images at the continual three largest ACSAs of the upper arm muscle between axillary cavity and cubital fossa were selected, and the average of these ACSAs was calculated (Popadic Gacesa et al. 2011). The CSAs of the skeletal muscle (SM) and the subcutaneous adipose tissue (SAT) were manually segmented by well-trained experts using image analysis software (SliceOmatic ver 4.3, Tomovision, Montreal, Canada). Bone tissue, connective tissue, and blood vessels were excluded from the measurements, wherever possible (Fig. 1).

To evaluate the validity of the MRI measurement, we tested the reproducibility of the intra-observer measurement, inter-observer reliability, and intra- and inter- day variability of image acquisition. The reproducibility of the intra-observer was estimated by the ICC. Analysis of the CSAs of SM and SAT by using same MR image was performed three times and the ICC(1,3) was calculated as described by McGraw and Wong (McGraw and Wong 1996). The inter-observer reliability was determined by the ICC(2,1), which compared the analyses of two observers (T.K. and M.Y.) from the same images of CSAs in all subjects. For testing the intra- and inter- day variability of image acquisition, 10 MR acquisitions were executed in two subjects. Intra- and inter- day variability of image acquisition were assessed by the coefficient of variation (CV), which is defined as the standard deviation divided by the average for the analysis of these images. After the subject had fasted for 10 h overnight the first measurement was performed before breakfast. Thereafter, the subject came repeatedly to the laboratory to be tested four times during 1 day: just before lunch, 2 h after lunch, just before dinner, and 2 h after dinner. Lunch and dinner was set at 12:00 and 19:00, respectively. This procedure was repeated on another day within a 1-week interval. Reproducibility of intra-observer measurement was very high; ICC(1,3) for area of SAT is 0.999, ICC(1,3) for area of SM is 0.999. High correlations were found in ICC(2,1), 0.919 and 0.937 for area of SAT and SM, respectively. Reproducibility of image acquisition of 10 times measurements was assessed as reliable by the CV for the 3.4% for SAT and 8.0% for SM.

### Data analysis

All data are presented as the mean ± S.D. The concordance correlation coefficient and Bland–Altman analysis with 95% limits of agreement was used to assess the agreement between BIA and MRI measurements. The unpaired t-test was used to assess the differences in physical characteristics and anthropological parameters between genders. The differences in segmental percentage of body fat between BIA and MRI measurements, the upper limb/upper arm and lower limb/thigh, and genders were analyzed using a three-way ANOVA with repeated-measures, and the Tukey–Kramer test was used for post hoc comparison. The level of statistical significance was set at *p* < 0.05.

## Results

**Fig. 2:**
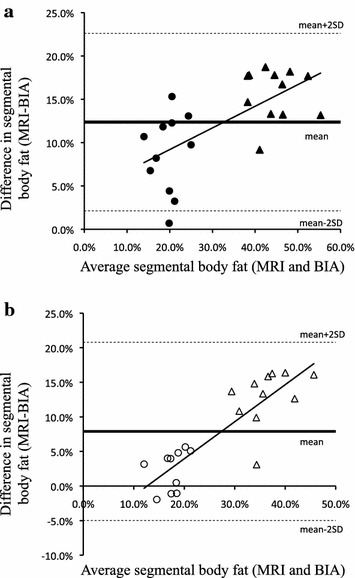
Bland-Altman plot showing individual error scores in segmental body fat of *upper limb* (**a**) and *lower limb* (**b**) measured by MRI and BIA in men (*circle*) and women (*triangle*).

Table 2 shows the percentage of segmental body fat in men and women obtained by BIA and MRI measurements. The values measured by the MRI method were significantly larger than those measured by BIA. BIA data showed that the percentage of fat was not different between the upper limb and lower limb for men; however, for women, the percentage of fat in the upper limb was significantly higher than that in the lower limb (p < 0.01). Contrary, MRI data showed that the percentage of fat in the upper arm was significantly higher than that in thigh for both men and women (p < 0.01). Gender differences were present in the fat percentage of the upper limb/upper arm and lower limb/thigh measured by both methods (p < 0.01). The concordance correlation coefficient between BIA and MRI measurements in upper limb/upper arm and lower limb/thigh were 0.863 (95% CI 0.715–0.937) and 0.562 (95% CI 0.224–0.779), respectively. Bland–Altman analysis showed that the mean difference between BIA and MRI measurements in upper limb/upper arm and lower limb/thigh were 12.4 ± 5.1% and 7.9 ± 6.4%, respectively, and that correlation coefficients between BIA and MRI measurements in upper limb/upper arm and lower limb/thigh were 0.660 and 0.862, respectively (Fig. 2).

**Table 2:** The segmental quantification of body fat in men and women obtained by BIA and MRI measurements

Body fat (%)	BIA		MRI	
	Men	Women	Men	Women
Upper limb/upper arm	15.6 ± 0.04	36.8 ± 0.06^#^	24.0 ± 4.3**	52.7 ± 5.9**^#^
Lower limb/thigh	16.4 ± 0.02	29.9 ± 0.04^#†^	18.9 ± 3.3^*†^	42.9 ± 5.8**^#†^

Values are presented as the mean ± SD.

* p < 0.05; ** p < 0.01 vs. BIA. ^#^p < 0.01 vs. Men. ^†^p < 0.01 vs. upper limb/upper arm.

**Fig. 3:**
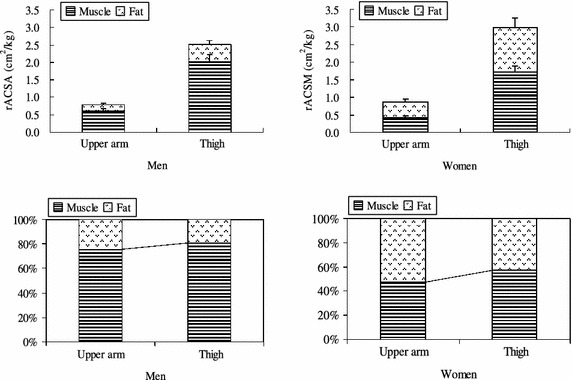
rACSAs (*upper panels*) and percentage (*lower panels*) of subcutaneous fat and skeletal muscle in upper arm and thigh for men and women obtained by MRI measurements.

Figure 3 shows the rACSAs and percentage of subcutaneous fat and skeletal muscle in upper arm and thigh for men and women. ACSAs of subcutaneous fat and skeletal muscle measured by MRI were 12.5 ± 3.5 and 38.4 ± 5.7 cm^2^ in upper arm and 32.1 ± 10.0 and 129.7 ± 15.7 cm^2^ in thigh for men and 23.5 ± 4.0 and 21.4 ± 4.8 cm^2^ in upper arm and 67.2 ± 15.7 and 88.2 ± 10.2 cm^2^ in thigh for women. When ACSAs were adjusted by body mass (relative ACSA: rACSA), rACSAs of subcutaneous fat and skeletal muscle were 0.19 ± 0.05 and 0.60 ± 0.06 cm^2^/kg in upper arm and 0.48 ± 0.11 and 2.03 ± 0.18 cm^2^/kg in thigh for men and 0.46 ± 0.08 and 0.41 ± 0.08 cm^2^/kg in upper arm and 1.29 ± 0.25 and 1.71 ± 0.2 cm^2^/kg in thigh for women. For both men and women, rACSAs of subcutaneous fat and skeletal muscle in upper arm were significantly lower than those in thigh; however, the amounts of subcutaneous fat relative to skeletal muscle in upper arm were significantly higher than those in thigh.

## Discussion

This study was the first to characterize the regional storage fat in the upper and lower limb of young adults as determined by BIA and MRI. According to Bland–Altman analysis, systematic trends were seen between BIA and MRI measurements in both upper limb/upper arm and lower limb/thigh. Indeed, the values measured by the MRI method were larger than those measured by BIA. With regards to men in this study, the BIA data showed that the percentage of fat in the upper limb was not different from that of the lower limb, while for women it was significantly higher than that of the lower limb. However, MRI data showed that the percentage of fat was significantly higher in the upper arm compared to the thigh for both men and women. Additionally, the percentage of fat in women was remarkably higher compared to men in both the upper limb and upper arm as measured by both BIA and MRI. These results revealed that BIA results in a significantly different estimation of the whole body and limb composition when compared to MRI and that the characteristics of the MRI method were useful in determining the regional specificities of the body composition of limbs.

Pathophysiological relevance to assess appendicular regional fat and muscle distribution would be revealed in the fact that greater fat mass distributed in appendicular sites, such as lower limbs, appears to be beneficial or protective against cardiovascular disease and/or metabolic risk factors as compared with central obesity (Hu et al. 2011; Park et al. 2014; Snijder et al. 2009). These protective effects of appendicular fat might be mostly attributed to the leg, especially thigh (Hunter et al. 2010; Park et al. 2014; Snijder et al. 2005). On the other hand, the study using DEXA to evaluate percentage of fat has shown less impact of upper limbs on diagnosing metabolic disease compared to lower limbs (Park et al. 2014). It is possible, however, that DEXA- or BIA-derived percentage of fat does not reflect site-specific fat and muscle distribution, thereby missing the association between regional limb composition and diagnostic criterion. Indeed, we found that the distribution of fat and skeletal muscle and hence percentage of fat fluctuated throughout the upper limb (data not shown). For instance, percentage of fat in forearm or upper arm assessed by a series of contiguous MRI volume analysis was lower or higher, respectively, compared to the average of percentage of fat in upper limb measured by BIA in the woman. Besides the inconsistency of site-specific percentage of fat between BIA and MRI, regional fatty infiltration, which can be measured by MRI, in skeletal muscle is associated with insulin resistance and other metabolic risk factors (Goodpaster et al. 2000; Zoico et al. 2010). More detailed measurements of site-specific fat and muscle distribution might be warranted to appropriately assess the relationship with the metabolic profile.

One of the interesting findings of this study was that the percentage of fat was significantly higher in the upper arm compared to the thigh in both men and women. The metabolic responses of the upper and lower limbs are likely to adapt differently through the evolutionary processes of human bipedality. Due to human hands being free from locomotion, the legs engage in motor activities. One of the key factors in the evolution of humans is the improved running economy that allows running longer at less metabolic cost (Bramble and Lieberman 2004). In quadruped locomotion, both the arm and leg contact the ground and were transferred a force from the trunk. The Pongo genus has the same or even larger forelimbs compared to hindlimbs (Morbeck and Zihlman 1988). It is believed that after humans became bipedal, the daily requirements on the force generating capacity of the arm muscles decreased and the size of the muscle became smaller than the lower limb. Additionally, the basal metabolic rate is lower when muscle volume is small and muscle activity is low. The morphological characters of the upper and lower limbs in humans could adapt differently over years after becoming bipedal. In the modern era, the increased inactivity has become a major cause of obesity and the metabolic syndrome. The frequency of muscle activation is a key for proper metabolic regulation. Because the total amount of muscle activity in daily life reflects muscle volume, the metabolic level of the upper and lower limbs should be different.

It is still unclear why an abundance of fat was stored in the upper arm. Local differences in adipose tissue storage in the upper and lower limbs can suggest regional metabolic responses in the body. It is well known that women have a higher percentage of body fat than men and that they store fat in the hip and thigh regions, as also seen in our results. This might be because the hip and thigh regions in women have a greater number of epinephrine alpha receptors, which inhibit lipolysis (Blaak 2001). Additionally, of most interest was the fact that the percentage of fat in both upper limbs as measured by BIA and the upper arm as measured by MRI in women was remarkably higher compared to men. Although the reasons for the large difference in the storage fat of the upper limb between men and women are not clear, it might be explained by hormonal differences. Men generally produce a greater amount of testosterone, which leads to greater muscularity. Additionally, it has been shown that the shoulder (trapezius) muscle has more androgen receptors than the leg (vastus lateralis) muscle (Kadi et al. 2000); this could lead to larger muscles in the neck, shoulder and upper arm. On the contrary, women do not produce a large amount of testosterone, but do produce a substantial amount of estrogen in order to develop female sexual characteristics in the hips and upper body by storing fat (e.g., breasts). Further studies should examine the regional lipid metabolic response under exercise conditions and how these acute responses are related to chronic metabolic adaptation or body composition between men and women.

There are several limitations in this study. Small number of subjects might be underpowered for some variables, and the subjects were only selected to young healthy Japanese. Also, confound factors such as daily physical activity levels and genetic background were not considered for body composition. Thus, further study is needed to specifically address the subgroups of age, ethnicity and physical fitness levels with a large sample size. Despite these limitations, our findings may provide an impetus to advance our understanding of physiological and biological significance of specific characterization in fat storage of human body.

## Conclusions

Our results suggest that BIA results in a significantly different estimation of the whole body and limb composition when compared to MRI and that MRI is useful to determine regional specificities in the limb composition. We found specific characterization in regional storage body fat of young healthy adults: there was a higher percentage of fat in the upper arm compared to the thigh in both men and women, and a higher percentage of fat in the limbs of women compared to men. Further studies are needed to elucidate the physiological and biological significance of these specific compositions.

## Abbreviations

ACSA: anatomical cross-sectional area
BIA: bioelectrical impedance analysis
CSA: cross-sectional area
CV: coefficient of variation
DEXA: dual-energy X-ray absorptiometry
ICC: intraclass correlation coefficient
MRI: magnetic resonance imaging
rACSA: relative ACSA (ACSA relative to body mass)
ROI: region of interest
SAT: subcutaneous adipose tissue
SM: skeletal muscle
